# Post‐Thrombotic Syndrome: Pathophysiology, Clinical Implications, and Advances in Management

**DOI:** 10.1002/hsr2.71656

**Published:** 2025-12-17

**Authors:** Than Htun, Rahulkumar Amrutiya, Kaung Htet Hla Win

**Affiliations:** ^1^ University of Medicine (1) Yangon Yangon Region Myanmar; ^2^ One Brooklyn Health/Interfaith Medical Center Brooklyn New York USA

**Keywords:** deep vein thrombosis, post‐thrombotic syndrome, venous hypertension

## Abstract

**Background and Aims:**

Post‐thrombotic syndrome (PTS) is a common and challenging complication of deep vein thrombosis (DVT). PTS is characterized by chronic venous insufficiency and persistent venous hypertension. Clinical features are nonspecific. Despite its high prevalence, PTS lacks standardized diagnostic criteria. Furthermore, PTS carries significant morbidity and puts a burden on the healthcare system, as the condition is irreversible and management options tend to be limited. Herein, we review the current literature on PTS regarding pathophysiology, diagnosis, and management.

**Methods:**

Articles regarding post‐thrombotic syndrome (PTS) were searched via PubMed, Embase, and Google Scholar between January 1980 and January 2025 using relevant keywords. Studies and reports written in languages other than English, for pediatric patients, and related to other venous disorders were excluded. Selected articles were reviewed in detail, and principal findings and outcomes of the studies were critically analyzed and summarized.

**Results:**

While diagnosis is largely clinical, scales such as the Villalta scale and CEAP classification provide tools for objective evaluation with limited accuracy and reliability. Current treatment options for PTS include targeted lifestyle modification, pharmacologic treatment, compression therapy, proper and timely anticoagulation, and interventions such as thrombolysis, thrombectomy, and stenting. Evidence is limited for compression therapy (stockings) currently; however, catheter‐directed interventions have stronger evidence of efficacy with several limitations in prevention and treatments.

**Conclusion:**

Overall, there is no definitive method to either entirely prevent PTS or effectively treat it once it is established. Preventing DVT remains the cornerstone of reducing PTS incidence. This review highlights the urgent need for comprehensive prevention strategies, improved diagnostic tools, and evidence‐based treatment protocols. Further research is essential to bridge the existing knowledge gaps, optimize patient outcomes, and develop effective approaches to both prevention and management.

## 1 Introduction and Background

### 1.1 Introduction

Post‐thrombotic syndrome (PTS) is one of the most common complications that may develop within the first 2 years after a lower limb DVT episode [1]. PTS is believed to result from a combination of venous hypertension due to thrombotic blockage and reflux because of valvular incompetence [2]. PTS can cause a wide set of symptoms ranging from minor clinical signs and symptoms, such as chronic pain in the leg, which interferes with activity or the ability to work, leg edema, and ulcers [3].

PTS has a significant impact on patient lives and healthcare systems [4]. Due to significant effects and increasing prevalence, venous thromboembolism and associated illnesses must be appropriately managed [5].

PTS often manifests 2 years following an episode of DVT; however, because standard diagnostic criteria are not available, incidence may vary from 20% to 63%. Currently available scoring systems that aid in the diagnosis are not standardized [6].

This review summarizes the existing knowledge of PTS in adults and assesses the risk factors, pathophysiology, diagnosis and treatment, and research gap.

### 1.2 Literature Search

A comprehensive literature search was performed using PubMed, Embase, and Google Scholar to identify relevant studies on PTS between January 1980 and January 2025. The search included keywords such as “post‐thrombotic syndrome,” “deep vein thrombosis sequelae,” “venous thromboembolism complications,” “PTS management,” and “chronic venous insufficiency after DVT.” Titles and abstracts were screened to exclude studies that were not relevant, publications in languages other than English, and articles focusing on pediatric populations or unrelated venous disorders. Reference lists of key publications were also reviewed to ensure inclusion of additional pertinent studies.

Eligible articles included observational studies, clinical trials, case reports, systematic reviews, meta‐analyses, and narrative reviews that addressed the pathophysiology, clinical manifestations, diagnosis, prevention, and management of PTS. Following removal of duplicates and non‐relevant studies, a total of 106 articles were included and analyzed to provide a comprehensive overview of the current understanding and advances in the management of PTS.

## 2 Review

### 2.1 Pathophysiology

PTS develops as a consequence of venous hypertension [3]. Two pathological processes underlie the development of venous hypertension: persistent venous obstruction and valvular reflux caused by damaged valves [7]. Acute DVT leads to venous obstruction, which can be partial or complete. DVT is followed by venous remodeling (as identified using ultrasound), which seems to have a clear connection to the thrombus [8]. Fibrosis of the affected vein wall with resultant luminal narrowing is the key underlying mechanism leading to PTS syndrome [9].

The occurrence of PTS has also been associated with increased levels of inflammatory cytokines or adhesion molecules, like IL‐6 and ICAM‐1, indicating that inflammation may be involved in the pathophysiology of PTS [10]. Wik et al. (2015) identified a statistically significant link between inflammatory markers, such as high‐sensitivity C‐reactive protein (hs‐CRP), and the development of PTS in pregnancy‐related DVT. Venous valves are directly damaged by the recanalization activity and the inflammatory reaction to acute thrombosis [11, 12, 13].

The probability of producing reflux is correlated with the degree of initial venous obstruction. Reflux develops early, and the degree of reflux progressively increases, affecting 17% of patients within 1 week and reaching 69% after 1 year following a DVT diagnosis [14].

Following DVT, recanalization is enabled by a combination of fibrinolysis, thrombus reorganization, and neovascularization. If there is a complete breakdown of the clot as a result of the above combination, it is associated with a lesser risk of PTS [15].

According to the current understanding of PTS, if there is incomplete lysis of the thrombus, it will lead to venous outflow obstruction, and this will lead to venous hypertension. This results in the calf muscle pump not working effectively, causing venous pressure to remain high during walking or exercise, which can lead to symptoms such as venous claudication, ankle swelling, skin changes, and ulcers [7].

**Figure 1:**
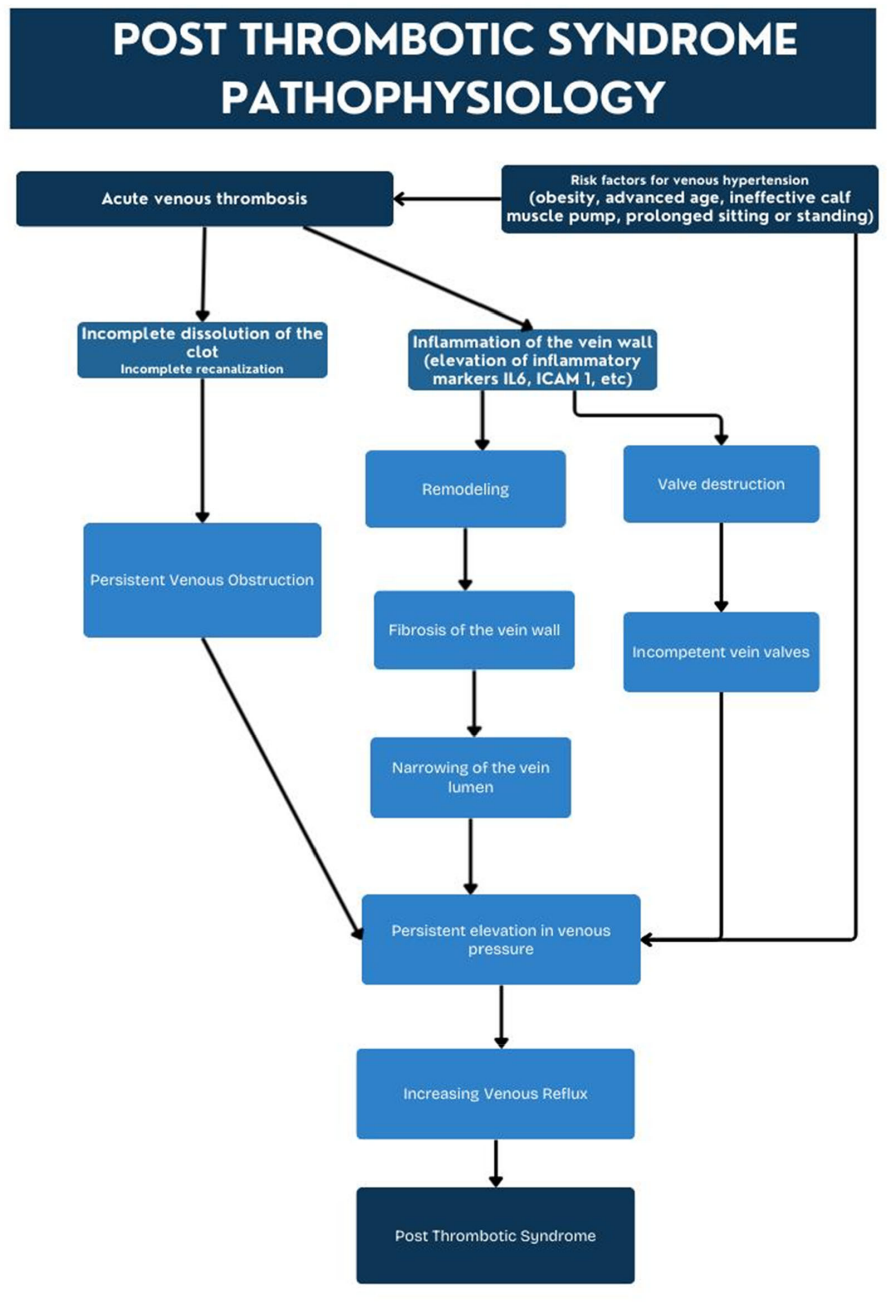
Pathophysiology of post thrombotic syndrome (PTS).

As with chronic venous insufficiency, the key driving factor for the development of PTS is the elevated venous pressure. Obesity and advanced age increase the risk of PTS, with the former increasing by twofold [16]. The pathophysiology of PTS is illustrated in Figure 1.

### 2.2 Incidence (Epidemiology)

PTS is the most prevalent complication of deep vein thrombosis (DVT). Typically, it is believed that between 20% and 50% of patients will develop PTS within 2 years following a DVT, with up to 10% experiencing severe PTS. In addition to having similar risk factors between PTS and chronic venous insufficiency, the incidence of PTS is higher in patients with underlying chronic venous insufficiency [16]. Nonetheless, the frequency of PTS as reported in studies can differ greatly. In addition to possible study biases (especially participants dropping out), other factors contributing to these variations include the types of diagnostic methods used for PTS evaluation, characteristics of the study group, the timeframe between initial DVT and PTS evaluation, and the frequency of PTS evaluations conducted [17].

### 2.3 Risk Factors for PTS

Several factors can contribute to the development of PTS. In general, older age and high BMI (> 30) increase the risk, while proximity of the clot and inadequate treatment of DVT with persistence of symptoms pose the chance of developing PTS substantially [3]. Risk factors for the development of PTS are tabulated in Table 1 [3, 16].

**Table 1:** Risk factors for development of PTS.

General risk factors	Older age Obesity History of previous ipsilateral DVT Underlying primary venous insufficiency
Risk factors at the time of diagnosis of DVT	Location of DVT (proximal DVT is more strongly associated with development of PTS) Efficacy of therapeutic anticoagulation (Risk of PTS increases with inadequate anticoagulation, e.g., with subtherapeutic INR in patients treated with warfarin)
Risk factors post‐DVT episode and apparent during the follow‐up period	Recurrence of DVT in ipsilateral limb Persistent symptoms and signs of venous insufficiency Evidence of residual thrombosis on ultrasonography 3‐6months after acute DVT Persistent elevation of d‐dimer

### 2.4 Clinical Features

Symptoms of PTS can start manifesting several weeks to months after an episode of DVT. They can be intermittent or chronic and may even continue uninterrupted after the acute incident [18].

The main complaints from patients are leg discomfort, such as heaviness or aching, throbbing or burning pain, itching, and swelling in the limb. Discomfort can be generalized throughout the leg (e.g., aching, burning) or localized to particular veins and their tributaries, regions of skin discoloration (hemosiderin buildup; either by itself or with inflammation), or regions of lipodermatosclerosis or ulcers. When severe clinical symptoms are present, walking may become challenging or even impossible due to pain [19].

Discomfort from venous disease tends to be intense while standing or sitting with feet down for a long time but gets better with walking and raising the limbs [20].

PTS patients report heaviness, restlessness, itching, swelling, varicose veins, venous skin alterations such as dermatitis and atrophie blanche, and ulceration as symptoms of chronic venous insufficiency. PTS frequently manifests with skin abnormalities such as lipodermatosclerosis, atrophie blanche, and venous eczema [7].

The changes result in thinning of the skin, which is prone to damage and frequently takes time to heal because of increased venous tension. Patients may also develop features of venous claudication, often described as intense discomfort relieved by resting and elevating the legs [7].

After a primary DVT episode, the edema of the lower limb might lessen; however, the limb may not return to its original dimensions. This swelling often worsens as the day progresses and is worse when standing for long periods. Skin alterations are typically the direct outcome of venous hypertension and can occur in conjunction with limb swelling [7].

### 2.5 Investigations

There is no definitive physiologic test, imaging, or biomarker to confirm the diagnosis of PTS. PTS is typically identified through clinical diagnosis when a patient who has had a previous DVT shows specific symptoms and signs [21]. It is recommended to wait until 3–6 months after the acute phase before diagnosing PTS to allow time for the initial pain and swelling from acute DVT to subside in certain patients [1]. PTS may manifest anytime between 3 and 6 months following DVT, or possibly up to 2 years later [22].

Elevated d‐dimer level might reflect persistent activation of clotting or inflammatory cascades. Although the specificity is poor, persistent elevation of d‐dimer in the absence of treatment with oral anticoagulant could signal recurrent venous thromboembolism [23]. Besides, the mean d‐dimer level was found to be significantly higher in patients who developed PTS than in those who did not in a 4‐month follow‐up [24].

For individuals with PTS, it is crucial to gather extensive details regarding the disease's pattern and the specific locations of occlusions or reflux zones. The initial stage of diagnostic evaluation involves a duplex ultrasound, which utilizes Doppler to search for reflux and flow, and a 2‐D ultrasound to look at vein anatomy. A duplex USG is beneficial because it provides real‐time imaging without invasive procedures, providing a comprehensive assessment of venous incompetence and obstructions. The downsides of this duplex ultrasound examination include the prerequisite for patients to stand for as long as 45 min per scanned leg and the challenge of visualizing the iliac and second‐rate vena cava (IVC) and venous frameworks, which may require extra modalities [7].

It has been seen that there is a correlation between the site of occurrence of DVT and the severity of PTS symptoms. Proximal lesions, such as iliofemoral ones, cause severe symptoms and have a higher chance of recurrence. Therefore, accurately determining the location of the clot is crucial in managing patients with PTS. However, it is important to emphasize that PTS is primarily a clinical diagnosis, and imaging primarily aids in identifying disease patterns and guiding further management [7].

### 2.6 Severity Scoring of PTS

The Villalta scale, shown in Table 2, was created for the assessment and diagnosis of PTS [1, 25]. An abstract was used to present the development and preliminary validation; however, a full‐text paper has yet to be published [21]. The Villalta scale is the most commonly used tool for diagnosing and assessing the severity of PTS in clinical studies. The International Society on Thrombosis and Hemostasis (ISTH) has approved the scale, and its growing number of reports highlights its validity [26, 27, 28].

**Table 2:** The villata scale.

Villata score				
Reported symptoms	None	Mild	Moderate	Severe
Pain	0	1	2	3
Cramps	0	1	2	3
Heaviness	0	1	2	3
Paresthesia	0	1	2	3
Pruritus	0	1	2	3
Clinical Signs	None	Mild	Moderate	Severe
Edema in pretibial area	0	1	2	3
Skin induration	0	1	2	3
Hyperpigmentation	0	1	2	**3**
Pain on compression of calf	0	1	2	3
Dilated or twisted veins (Venous ectasia)	0	1	2	3
Readness	0	1	2	3
Venous ulcer	0 if absent			3 if present
Score interpretation				
0–4	No disease present (negative for PTS)			
5–9	Mild			
10–14	Moderate			
15 or more	Severe			

The Villalta score shows strong interobserver repeatability [29], strong correlations with other classification systems [30], and strong correlations with quality‐of‐life indicators [25]. The ISTH has advised using the Villalta score to determine the existence and severity of PTS [1].

The CEAP classification was introduced by the American Venous Forum with the purpose of classifying and evaluating treatment outcomes in patients with chronic venous disease (CVD) [31, 32, 33, 34]. This is illustrated in Table 3 [33, 34].

**Table 3:** CEAP classification.

Clinical signs	C0 ‐ No visible or palpable signs of venous disease
	C1 ‐ Telangiectasia or reticular veins (diameter < 3 mm)
	C2 ‐ Varicose veins (diameter of ≥ 3 mm)
	C3 ‐ Edema
	C4 ‐ Changes in skin and subcutaneous tissue secondary to chronic venous disease
	C5 ‐ Healed venous ulcer
	C6 ‐ Active venous ulcer
	S‐ Symptomatic
	A‐ Asymptomatic
Etiology	Ec‐ Congenital
	Ep‐ Primary
	Es‐ Secondary
	En‐ No cause identified
Anatomy	As‐ Superficial veins
	Ap‐ Perforating veins
	Ad‐ Deep veins
	An‐ No venous location identified
Pathophysiology	Pr‐ Reflux
	Po‐ Obstruction
	Pr,o‐ Reflux and obstruction
	Pn‐ No venous pathophysiology identifiable

*Note:* C (Clinical Signs), E (Etiology), A (Anatomic Distribution), P (Pathophysiology) Classification.

According to the CEAP classification, patients are classified based on clinical symptoms (C0–C6), which is most common; they can also be classified based on underlying cause, anatomical extent of venous disease, and pathophysiology, i.e., if the venous incompetence is either caused by obstruction or reflux or both. Additionally, patients can be categorized into symptomatic or asymptomatic [35].

The CEAP classification considers the disease pattern, not the severity reported by the patient. CEAP is valuable as a research tool when studying individuals with chronic venous disease [7].

The venous clinical severity score (VCSS) was developed and validated by the American Venous Forum using data from the national venous screening program in the United States. It serves as a valuable tool for assessing disease severity and monitoring treatment progress, as tabulated in Table 4 [7].

**Table 4:** The venous clinical severity score.

The venous clinical severity score				
	None: 0	Mild: 1	Moderate: 2	Severe: 3
Pain or discomfort	None	Occasional pain or other discomfort	Daily pain or other discomfort	Daily pain or discomfort
Varicose veins varicose veins > 3 mm in diameter to qualify for the standing position	None	Few: scattered Also includes corona phlebectatica (ankle flare)	Confined to calf or thigh	Involves calf and thigh
Venous edema	None	Limited to foot and ankle area	Extends above the ankle but below knee	Extends to knee and above
Skin pigmentation	None or focal	Limited to perimalleolar area	Diffuse over the lower third of calf	Wider distribution above the lower third of the calf
Inflammation	None	Limited to perimalleolar area	Diffuse over the lower third of calf	Wider distribution above the lower third of the calf
Induration	None	Limited to perimalleolar area	Diffuse over the lower third of calf	Wider distribution above the lower third of the calf
Number of active ulcers	0	1	2	> 3
Duration of active ulcer (longest active)	N/A	< 3 months	> 3 months but < 1 year	Not healed for > 1 year
Size of largest active ulcer	N/A	Diameter < 2 cm	Diameter 2–6 cm	Diameter > 6 cm
Use of compression therapy	0; Not used	1; Intermittent use of stockings	2; Wears stockings most days	3; Full Compliance: stockings

The VCSS was designed to be a more straightforward tool than the CEAP classification for patients with CVD and is adjustable to changes over time [36]. Although the VCSS was recently updated, its effectiveness in diagnosing and grading PTS has not been proven [37]. There are only a few studies that investigated the VCSS for PTS [38, 39, 40, 41]. Although the VCSS has shown the ability to differentiate between a leg with a history of DVT and the contralateral leg in terms of its specificity for PTS, some patients with normal ambulatory venous pressure have been diagnosed with severe PTS [30]. There is no research examining correlations between Health‐related Quality of Life (HRQoL) and PTS identified by the VCSS.

The characteristics of the Villalta scale, CEAP classification, and the VCSS are summarized in Table 5 [31].

**Table 5:** A comparison of diagnostic scoring systems for PTS.

	The villalta scale	CEAP classification	The venous clinical severity score
Inter‐observer reliability	Yes	No evidence for PTS	No evidence for PTS
Validity	Yes	No standardization for diagnosis of PTS	Yes
Assessing the severity of PTS	Yes	Difficult to quantify PTS severity	Yes
Strength	Most widely used scoring tools for PTS diagnosis, severity assessment, and follow‐up	Categorization and reporting of chronic venous diseases	Ability to objectively grade the severity and to assess change over time/with treatment
Weakness	Limited accuracy in diagnosis of PTS (e.g., with previous chronic venous disease)	Alphabetical elements leading to subjectivity and difficulties in severity grading	Poor correlation with other scoring systems

### 2.7 Prevention and Treatment

Given that PTS is a consequence of DVT, the most effective approach to avoid it appears to be to prevent DVT in individuals who are at risk and reduce the likelihood of having DVT again following an initial incidence [36]. Chronic venous insufficiency is a major risk factor for the development of DVT. Thus, early and effective treatment of chronic venous insufficiency would perhaps reduce the incidence and complication of DVT, including PTS. Numerous research studies and guidelines [42, 43, 44, 45] emphasize the importance of maximizing the effectiveness of primary or secondary thrombosis prevention in high‐risk scenarios. Once diagnosed, the management of PTS is challenging in part due to post‐thrombotic fibrosis with remodeling of the affected vein leading to complete luminal obstruction [46].

Studies have shown that inadequate anticoagulation with vitamin K antagonists within the first 3 months after a DVT episode can increase the risk of developing PTS [47, 48]. To reduce the risk of PTS, strict INR monitoring is therefore advised, especially during the initial 3 months of therapy [49]. However, the risk of developing PTS cannot be eliminated by antithrombotic therapy; individuals receiving optimum anticoagulation still have the potential to develop this condition.

Low molecular weight heparins (LMWH) are suggested to be better than vitamin K antagonists (VKA) in preventing PTS, partly due to their anti‐inflammatory properties [50]. Some studies have shown reductions in venous ulcers and improved vein recanalization with LMWH compared to VKA [51]. Additionally, fewer patient‐reported PTS symptoms were noted with LMWH [52, 53]. However, these studies have significant limitations, such as using non‐validated methods for PTS diagnosis and assessing PTS too early, when symptoms may not fully develop [1, 52, 54, 55, 56, 57]. Consequently, the sole use of LMWH for preventing PTS is still a subject of debate [58, 59].

The effectiveness of direct oral anticoagulants (DOACs) compared to VKA for PTS prevention is still uncertain [60]. A post‐hoc analysis of one study did not find a significant difference between rivaroxaban and a combination of low‐molecular‐weight heparin (LMWH) and vitamin K antagonists (VKA) [61]. Further trials are needed to determine the role of DOACs in PTS prevention.

### 2.8 Exercise

Physiologically, exercise may contribute to the development of collateral blood flow and enhance the pumping action of the calf muscles, thereby reducing the risk of PTS. Alternatively, increased muscle blood flow to an already obstructed and malfunctioning venous system could worsen venous pressure. Early mobilization and compression have been shown to significantly reduce the risk of PTS. On the other hand, a study involving 387 patients did not find a link between the level of physical activity 1 month after DVT and the occurrence of PTS. Additionally, structured exercise programs following DVT have not been shown to improve the recanalization of blocked veins [60]. Other lifestyle measures, such as weight loss and leg elevation, appear to improve the symptoms of PTS [62]. Studies pointed out the strong correlation between obesity and the CEAP clinical stage of venous disease, possibly due to raised intra‐abdominal pressure resulting in greater reflux, increased vein diameter, and venous pressures [63]. Meanwhile, elevation of limbs while resting has been found to improve microcirculatory flow by reducing hydrostatic pressure in the veins [64].

### 2.9 Compression Therapy

Elastic compression bandages/stockings (ECS) are used after an acute DVT to prevent PTS as well as to ameliorate venous symptoms [65]. Applying external compression early on may prevent PTS by reducing fluid leakage from the blood vessel (decreasing transcapillary filtration), increasing fibrinolysis, and stimulating the formation of collaterals [66]. Previous randomized trials pointed out the reduction in the incidence of PTS by approximately 50% with the application of ECS therapy [67, 68]. Nonetheless, current practices vary widely, with no proper agreement on the optimal timing, duration, pressure, or length of compression bandages. Additionally, only one‐third of healthcare providers frequently prescribe compression bandages to asymptomatic patients to prevent PTS [69].

The idea that daily use of ECSs can prevent PTS has been disproved by several RCTs [70, 71]. A multicenter randomized placebo‐controlled trial comparing active use of ECS for 2 years versus placebo after an episode of proximal DVT demonstrated no statistically significant benefit of ECS in prevention of PTS [26]. Furthermore, another randomized controlled trial failed to reveal the evidence regarding the use of ECS in reducing the risk of PTS defined using Villalta‐Prandoni Score and VCSS instruments [70]. The discrepancy of the results of these trials appeared to be related to the placebo effect and/or lack of optimal compliance [71].

By pooling results from various independent randomized trials, a nonsignificant reduction in the risk of PTS was observed when comparing ECS with placebo [72]. Thus, American Society of Hematology guidelines and the CHEST Guideline and Expert Panel Report currently recommend against routine use of ECS to prevent the occurrence of PTS [72, 73]. However, a trial of ECS can be considered to improve symptoms [73].

Although the overall benefit appears to be modest, venous‐return assist devices (VRAD) can be considered in a subset of patients with moderate to severe PTS with refractory symptoms or if they cannot wear ECS. VRAD is a type of compression pump designed to increase circulation in the lower limbs. A two‐center, randomized, crossover‐controlled trial demonstrated a significant reduction in the mean Villalta scale score in patients using a VRAD device (Venowave) compared to the control at the end of the 8‐week study period [74].

### 2.10 Pharmacologic Treatment

The goal of pharmacologic treatment is to lessen the pathophysiologic mechanisms that underlie venous hypertension and inflammation [75].

Currently, there are limited pharmaceutical options for treating PTS, including rutosides, defibrotide, and hidrosmin.

Rutosides are flavonoid compounds found in many plants, and an herbal medication derived from them is well‐known and widely used for treating edema associated with venous insufficiency [76]. Although its exact mode of action is uncertain, it appears to be related to reducing capillary filtration and permeability to proteins [77]. A study found that (beta‐hydroxyethyl)‐rutoside treatment improved tiredness symptoms but had little effect on calf size after 8 weeks [78, 79]. The study was limited by a small sample size and short follow‐up. An observational study reported that rutosides reduced limb size, swelling, the need for invasive treatments, and ulcers in PTS patients. However, due to its non‐randomized design, the results should be interpreted cautiously [79].

Defibrotide, a complex of phosphodiester oligonucleotides from porcine intestinal mucosa [79], has unclear mechanisms but is thought to increase prostacyclin, prostaglandin E2, and thrombomodulin, reduce plasminogen activator inhibitor‐1 [77], and prevent endothelial injury [80]. It is approved by the FDA for preventing and treating sinusoidal veno‐occlusive disease post‐bone marrow transplantation [81]. Its use in PTS is less established. A study showed that defibrotide was better than placebo in reducing ankle swelling, pain, edema, and the recurrence of DVT over 12 months [82]. Notably, wearing ECS was a requirement for every research participant. Further, PTS was not diagnosed in compliance with current criteria and was not identified as a key end goal of the research [83].

Hidrosmin is also a flavone [84] whose exact mechanism of action is unclear [77]. There is limited data on its usage in treating PTS. A trial compared hidrosmin and rutosides over a 12‐month treatment period, with 6 additional months of follow‐up. Both drugs improved PTS symptoms, with hidrosmin showing slightly better results during treatment. However, the benefits disappeared after stopping the therapy [85].

While some studies have suggested potential benefits of certain medications for PTS, the evidence is generally limited and of low quality [77, 86]. A meta‐analysis found no significant advantage of rutosides over no treatment or elastic compression stockings (ECS) [86]. Given their short treatment duration and limited global approval, these drugs are not routinely recommended. Their use might be considered mainly for patients who haven't responded to other treatments.

### 2.11 Thrombolysis, Thrombectomy, and Stenting

Chronic venous outflow blockage is increasingly being treated with endovascular therapy, which frequently involves venous stenting. This is especially true for bigger veins like the iliocaval system [7]. If there is a blockage in venous outflow, it is treated first. Treatment options for obstructed iliac vein segments include endophlebectomy, venous bypass, and percutaneous angioplasty with or without stenting [87, 88, 89, 90, 91, 92, 93]. A randomized controlled trial looking at the effect of venous stenting for iliofemoral deep vein obstruction compared with the control group in patients with unilateral PTS revealed statistically significant improvement in terms of disease‐specific quality of life, pain disease index, and VCSS [94]. Looking at surgical reconstruction, meanwhile, strong evidence supporting its routine use to treat PTS is lacking [95]. Thus, endovascular techniques should be considered as the first treatment except in major disease with no other treatment options (e.g., C4‐C6 disease), severe PTS, and patients with disabling venous claudication with marked limitation of activities [95]. For patients with severe proximal reflux, surgical venous bypass can be considered, but only after percutaneous interventions have failed to restore the flow. This procedure is only available at specific high‐volume referral facilities [93, 96, 97, 98]. However, the need for technical expertise, careful patient selection, and consideration for complications limit the employment of both interventions in practice.

Although previous studies failed to show remarkable benefits of catheter‐directed thrombolysis (CDT) in reducing PTS incidence, newer randomized controlled trials comparing proximal DVT with control groups such as CAVENT and ATTRACT have shown reductions in the occurrence of PTS and symptom severity of PTS (iliofemoral group only), respectively [99]. It is crucial to note that recently developed clots may respond better to thrombolytic therapy, and thus, time to CDT would be an important factor in preventing PTS [100]. A systematic review and meta‐analysis of RCTs comparing CDT along with traditional anticoagulation versus anticoagulation alone for adult patients with DVT found a statistically significant reduction in risk of overall PTS with a number needed to treat of 10 (the severity determined by the Villalta score) in the treatment group with both CDT and anticoagulation [101]. Furthermore, the analysis revealed no difference in the rate of death or bleeding among the population studied. On the other hand, a recent Cochrane review evaluating systemic, locoregional, CDT, and pharmacomechanical thrombolytic therapies indicated that thrombolysis in general reduced the risk of PTS without any statistically significant risk reduction in the CDT group [102]. Of note, pooled analysis of data for both systemic and local thrombolysis with CDT in this study exhibited a statistically significant increased risk of bleeding related to such intervention [102]. The increased risk of bleeding associated with lytic catheter‐based interventions (thrombolysis with or without mechanical assistance) performed for early thrombus reduction in acute proximal DVT was also noted in a recent meta‐analysis, although there was a statistically significant benefit of reducing the risk of PTS [103].

Therefore, to consider CDT to treat or prevent PTS, it is important to take into account the potential benefits and risks of CDT, taking into account key factors such as severity of DVT (e.g., patients' symptoms, comorbidities, extent of venous system involvement), patients' activity levels, duration of DVT, and risk of bleeding, along with careful and diligent patient selection [101].

Traditional management of DVT of the lower extremities with anticoagulation alone was not sufficient to prevent recurrence and the development of PTS. While minimizing the risks of bleeding complications, percutaneous mechanical thrombectomy (PMT) appeared to be a safer and effective treatment approach for acute iliofemoral DVT. In the meta‐analysis study comparing PMT and CDT alone for management of acute iliofemoral DVT, pooled data suggested a statistically significant role of PMT in restoration of venous patency, prevention of DVT recurrence, and PTS while offering a reduced risk of bleeding complications [104]. Furthermore, a single‐center, retrospective cohort study pointed out that venous leg ulcers (VLU), a serious complication of PTS due to secondary post‐thrombotic obstructions, can be treated with mechanical thrombectomy, resulting in complete or near‐complete VLU healing within a few months of treatment [105]. Another retrospective observation study conducted in the department of plastic and microvascular surgery at a tertiary care hospital over 5 years also revealed the fact that thrombectomy is an effective and reliable therapeutic modality for DVT patients in terms of regaining venous patency, preventing recurrence of DVT and pulmonary embolism, and treating PTS [106]. Considering this evidence, mechanical thrombectomy is a promising approach in the prevention and treatment of PTS.

Key characteristics of studies included in the paper are summarized in Table 6.

**Table 6:** Key studies on post‐thrombotic syndrome (PTS).

Author (year)	Study design/population	Sample size/setting	Main focus	Key findings
Kahn et al. (2008)	Prospective cohort, DVT patients	*n* = 387, Canada	Risk factors	Higher BMI, recurrent DVT, and proximal vein involvement increased PTS risk
Prandoni et al. (1996)	Prospective study, first DVT	*n* = 355, Italy	Natural history	PTS developed in ~30% at 8 years
Brandjes et al. (1997)	RCT, compression stockings versus none	*n* = 194, Netherlands	Prevention	Stockings reduced PTS incidence by ~50%
Villalta et al. (1994)	Clinical scoring validation	*n* = 180, Italy	Diagnosis	Introduced Villalta scale for standardized PTS diagnosis
Kahn et al. (2014, SOX trial)	RCT, compression stockings versus placebo	*n *= 803, Canada	Treatment/prevention	No difference between groups; challenged routine stocking use
Jayaraj, A., & Meissner, M. (2015)	RCT, compression stockings versus placebo	*n *= 69	Treatment/prevention	Use of graduated compression stockings does not reduce incidence of PTS
Shekarchian et al. (2023)	RCT, Best medical thearpy (BMT) with stenting versus BMT alone	N = 63	Treatment/prevention	Symptomatic patients with deep vein obstruction have a better quality of life with BMT and stenting
Mastoris et al. (2019)	Meta‐analysis, Catheter‐directed thrombolysis versus anticoagulation	*n* = 1005	Treatment/prevention	Catheter‐directed thrombolysis reduces rates of PTS and improves iliofemoral vein patency
Broderick et al. (2021)	Meta‐analysis, Cochrane review, thrombolysis (with or without adjunctive clot removal strategies) and anticoagulation versus anticoagulation alone	*n* = 1943	Treatment/prevention	Thrombolysis in general along with anticoagulation, decreases the rate of PTS while increasing the risk of bleeding
Wong et al. (2019)	Systemic review, percutaneous mechanical thrombectomy (PMT) versus catheter‐directed thrombolysis (CDT)	*n* = 1170	Treatment/prevention	PMT was associated with a lower risk of PTS and bleeding complications
Philip et al. (2024)	A retrospective study on the efficacy of thrombectomy on PTS	N = 451 (India)	Treatment/prevention	Thrombectomy can restore venous patency and improve features of PTS

## 3 Conclusions

PTS is a common and serious complication of DVT, caused by venous hypertension from persistent obstruction and valvular incompetence, leading to chronic symptoms such as pain, swelling, and skin changes that significantly impair quality of life. Despite optimal anticoagulation, PTS remains challenging to diagnose and treat. Current diagnostic strategies, such as the use of the Villalta scale, rely on subjective assessments, and there is a lack of gold standard investigation. However, future use of objective methods (e.g., Magnetic Resonance Venography or intravascular ultrasound) and biomarkers may improve accuracy and guide therapy. Treatment options, including compression therapy, exercise, and certain pharmacologic agents, show limited benefits, while more invasive interventions, such as thrombolysis, stenting, and thrombectomy, have stronger evidence of efficacy with several limitations. This emphasizes the ongoing need for further research and clinical trials into the pathophysiology, diagnostic approach, and therapeutic interventions to prevent and address the debilitating features of PTS.

## Author Contributions

**Than Htun:** conceptualization, writing – original draft, methodology, visualization, writing – review and editing. **Rahulkumar Amrutiya:** conceptualization, methodology, writing – original draft, visualization. **Kaung Htet Hla Win:** conceptualization; methodology; writing – review and editing; writing – original draft.

## Funding

The authors have nothing to report.

## Conflicts of Interest

The authors declare no conflicts of interest.

## Transparency Statement

The lead author Than Htun affirms that this manuscript is an honest, accurate, and transparent account of the study being reported; that no important aspects of the study have been omitted; and that any discrepancies from the study as planned (and, if relevant, registered) have been explained.

## Data Availability

Data sharing is not applicable to this article as no new data were created or analyzed in this study.
